# *Imperata cylindrica*: A Review of Phytochemistry, Pharmacology, and Industrial Applications

**DOI:** 10.3390/molecules26051454

**Published:** 2021-03-07

**Authors:** Young-Kyung Jung, Dongyun Shin

**Affiliations:** College of Pharmacy, Gachon University, 191 Hambakmoe-ro, Yeonsu-gu, Incheon 21936, Korea; suntnrud@naver.com

**Keywords:** *Imperata cylindrica*, edible medicinal herb, glycosides, immunomodulatory, antitumor

## Abstract

*Imperata cylindrica* is a medicinal plant native to southwestern Asia and the tropical and subtropical zones. To date, 72 chemical constituents have been isolated and identified from *I. cylindrica* Among these compounds, saponins, flavonoids, phenols, and glycosides are the major constituents. Investigations of pharmacological activities of *I. cylindrica* revealed that this edible medicinal herb exhibits a wide range of therapeutic potential including immunomodulatory, antibacterial, antitumor, anti-inflammatory, and liver protection activities both in vivo and in vitro. The purpose of this review is to provide an overview of *I. cylindrica* studies until 2019. This article also intends to review advances in the botanical, phytochemical, and pharmacological studies and industrial applications of *I. cylindrica*, which will provide a useful bibliography for further investigations and applications of *I. cylindrica* in medicines and foods.

## 1. Introduction

The genus *Imperata cylindrica*. a member of Gramineae family, is a perennial rhizomatous plant that can grow on soils with a vast range of nutrients and moisture [1]. It is widely distributed in southwestern Asia and is specifically native to the tropical and subtropical zones. The dried rhizomes of *Imperata cylindrica* have been commonly used in China named “Bai mao gen”, in Korean named Mo-Geun-Chu-Chul-Mul, and in Japan as a crude drug named “Boukon (*Imperata rhizome)*”. The existing literature indicates that these herbal plants could be taken both internally and externally and it is regarded as a safe and effective treatment strategy. The rhizome of *Imperata* has been recorded in multiple versions of Chinese Pharmacopoeia, at the same time, it is also traditionally and ethnically used as a medicinal herb in Japan and Korea. In traditional Chinese medicine (TCM), the rhizome of *Imperata* could be used either alone or in combination with other herbal medicines to cure hematuresis, jaundice with damp-heat pathogen, emesis, hemorrhage and reducing fever and causing diuresis, anti-fever, and anti-inflammatory. A variety of classical formulas containing *I. cylindrica* is widely used in TCM clinic. For instance, a combination of *I. cylindrica*, *Panax quinquefolius*, *Rehmannia glutinosa*, and *Wolfiporia cocos* is commonly used for the treatment of hematuresis. The combination of *I. cylindrica*, *Artemisia capillaris* Thunb, and *Gardenia jasminoides* Ellis could significantly dispels dampness and heat. Thus, this is used widely in jaundice with damp-heat pathogen.

**Figure 1:**
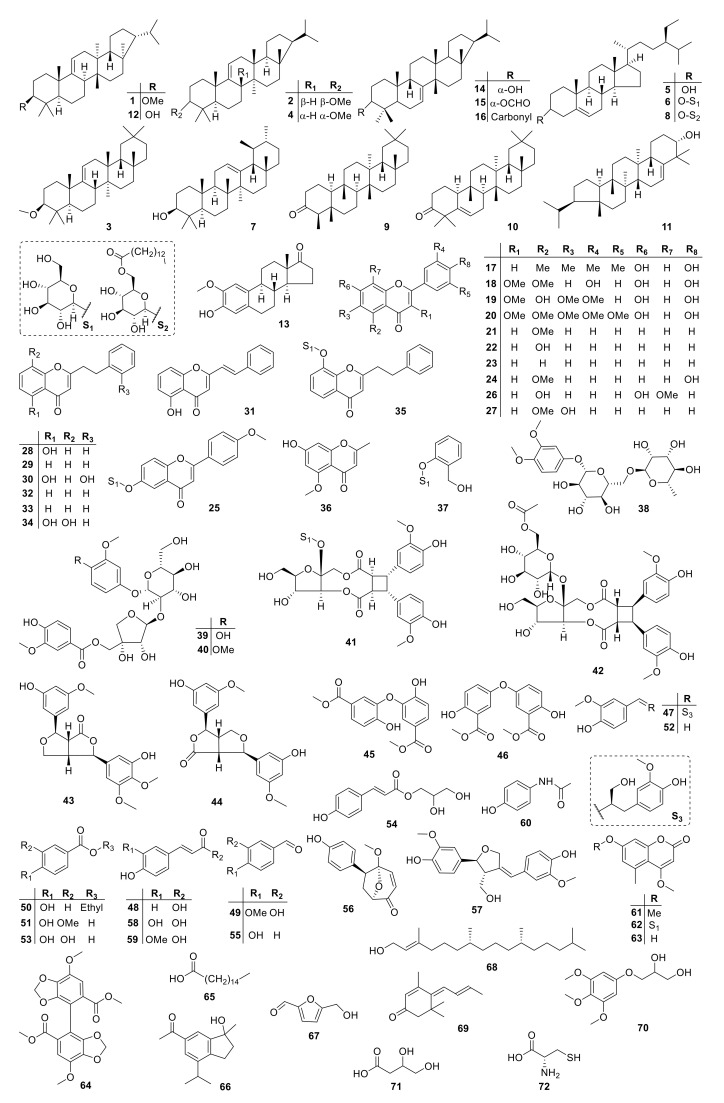
Constituents isolated from *I. cylindrica.*

Until now, a total of 72 compounds have been isolated and identified from the *I. cylindrica* plants, and the major phytochemical constituents identified in *I. cylindrica* are saponins, glycosides, coumarins, flavonoids, and phenols. (Figure 1). Modern pharmacology investigations on *I. cylindrica* have indicated that several substances from *I. cylindrica.* exhibit a wide range of biological activities such as hemostasis, improvement of urination, anti-inflammatory, antibacterial, anticancer, enhancement of the immune system, etc.

In this review, we intended to systematically present the advances achieved in phytochemical studies in recent decades, along with all the compounds listed. Additionally, we now provide a systematic overview of the available information of *I. cylindrica* including traditional uses, botany, nutritional composition, physiochemical, pharmacology, and industrial use.

## 2. Botany

### 2.1. Botanical Traits

*I. cylindrica* is a member of Gramineae family. According to “The Plant List” (www.theplantlist.org, Dec. 2019) *I. cylindrica* is the only accepted name for the plant with relative to other synonyms including *I. cylindrica* var. *Africana* (Andersson) C.E.Hubb., *I. cylindrica* var. *condensata* (Steud.) Hack., *I. cylindrica* var. *cylindrica*, *I. cylindrica* var. *europaea* (Andersson) Asch. & Graebn., etc.

*I. cylindrica* is a perennial herb about 25–120 cm tall. Its culms often have 1–4-noded and its tough and scaly rhizomes spread widely. The leaf blades of *I. cylindrica* are often stiffly erect with a flat or rolled shape. The margins of culm blades are scabrid with pubescence on its adaxial surface. The flowers of *I. cylindrical* are panicle cylindrical with copious numbers of hairs. The spikelets are about 2.5–6 mm long. Glumes with long silky hairs on the back have 5–9-veined. The length of the lower ciliate ovate-lanceolate lemma is two-thirds of a glum and the length of the denticulate ciliate upper lemma is one half of a glum. The anthers are about 2.2–4 mm with purplish black color.

### 2.2. Traditional Uses

Generally, *I. cylindrica* plants have been used in traditional systems of medicine in oriental area. It was first recorded in the Chinese medical classic “Shen Nong’s herbal Classic”(Han Dynasty), which is listed as a middle grade herbal. It is described sweet in taste, cold in nature, and attributive to the lung, stomach, and bladder meridians, with relieving fever, and a diuretic effect. For the treatment of fever, polydipsia, vomiting blood, blood stasis, lung heat, difficulty in micturition, edema, jaundice, etc. Based on “Shen Nong’s herbal Classic (Han Dynasty)” *I. cylindrica* was used to invigorating spleen and replenishing qi. Complying with “Ming Yi Bie Lu” (Han Dynasty), *I. cylindrica* was used to irregular menses. According to “Ben Cao Gang Mu” (Ming Dynasty) the main function of *I. cylindrica* was to diuresis to eliminate edema, hemostasis. In “Ben Jing Feng Yuan” (Qing Dynasty), the *I. cylindrical* was described as a treatment for frequent vomiting. The usual processing methods of *I. cylindrical* are decocting with water, and *I. cylindrical* is also considered for external use. A therapy dosage of 9–30 g of *I. cylindrica* has been recommended in the 2015 edition of Chinese Pharmacopoeia.

For the purpose of compatibility of medicines, *I. cylindrica* was typically matched with the *Morus alba L.* for treatment of asthma. Moreover, *I. cylindrica* can be used in a combination with *Puerariae lobatae* Radix to relieve high fever with sweet. Besides, *I. cylindrica* and *Rhizoma phragmitis* can be used to lower the negative Qi to stop vomiting.

## 3. Nutritional and Physiochemical Composition

### 3.1. Nutritional Composition

Apart from above constituents, nutrient substances including crude fiber, carbohydrates, sugars, fatty acid, and trace elements have been proved to be contained in the *I. cylindrica*, indicating the low-calorie and health-promoting properties of *I. cylindrica*. The carbohydrates and sugars are energetic sources and add flavor to plants, whose presence reveals the potential of *I. cylindrica* as functional foods [2]. The inorganic nutrient analysis of the aboveground living parts of *I. cylindrica* showed that Ca^2+^, Na^+^, Mg^2+^, Fe^3+^, and K^+^ are the main trace elements in the plant [3]. Moreover, *I. cylindrica* can survive in seawater salt concentrations and simultaneously have high nutraceutical potential, for instance, some of these species have already been considered edible for cattle [2].

### 3.2. Physiochemical and Structural Features

The detailed phytochemical and nutritional analyses of *I. cylindrica* had been carried out. Among the constituents isolated from *I. cylindrica*, saponins, glycosides, flavonoids, coumarins, and phenols are the primary types. All compounds are summarized and compiled in Table 1, and the structure of them has been detailed in Figure 1. 

#### 3.2.1. Saponins

*I. cylindrical* contain multiple triterpenoids. To date, 16 triterpenoid compounds have been isolated from *I. cylindrica* (**1**–**16**). Among them, most of compounds are pentacyclic triterpenoid sapogenins except for compounds (**5**), (**6**) and (**8**). The basic skeleton of the pentacyclic triterpenoid sapogenins could be divided into fernane and arborane. Arundoin (**1**) and cylindrin (**2**) are the first set of compounds isolated from *I. cylindrica,* which were methyl ethers of fernane type pentacyclic triterpenoid sapogenins, and friedelin (**9**) is an arborane type pentacyclic triterpenoid saponin. Additionally, some other triterpenoids such as 14-epiarbor-7-en-3β-ol (**14**), 14-epiarbor-7-en-3β-yl formate (**15**), and 14-epiarbor-7-en- 3-one (**16**) could also be found in this category. The contents of the triterpenoids are shown in Table 1.

#### 3.2.2. Flavonoids

Flavonoids are a typical category of compounds present in the *I. cylindrica* plants. Among them, mainly chromone and other characteristic structure of flavonoids (**17**–**36**).

The chromone **28**, **29**, **32**, and **36** have been isolated from *I. cylindrica*. Compound **28** showed significant neuroprotective activity against glutamate-induced neurotoxicity at 10.0 µm concentration in primary cultures of rat cortical cells (Jeong Seon et al., 2006). Some characteristic flavonoids have been separated and identified from *I. cylindrica* as well, such as tricin (**17**)**,** caryatin (**18**), jaceidin (**19**), 5-methoxyflavone (**21**)**,** 5-hydroxyflavone (**22**), flavone (**23**)**,** and so on.

#### 3.2.3. Glycosides

Biologically important glycosides have been less widespread in the *I. cylindrica*. To date, 6 types of glycosides compounds (**37**–**42**) have been isolated from the plants including impecyloside (**42**) as a lignin glycoside, seguinoside K (**39**), gymnetinoside D (**40**), and deacetylimpecyloside (**41**).

#### 3.2.4. Phenols

Phenols are widely distributed in *I. cylindrica*. Approximately 18 kinds of phenols (**43**–**60**) have been isolated from *I. cylindrical*. Among them, simple phenols and phenolic acids can be found such as vanillic acid (**51**), caffeic acid (**58**), ferulic acid (**59**), protocatechuic acid (**53**), and so on. Moreover, the phenylpropanoids 1-O-p-Coumaroylglycerol (**54**) and biphenyl ether cylindol A (**45**) and cylindol B (**46**) also have been isolated from *I. cylindrica*. Along with the lignans graminone A (**44**) and graminone B (**43**), where graminone B at 10^−4^ M gave a 50% inhibition of the contractile response of the aorta isolated from rabbit to KCI (30 mM). Imperanene (**47**) exhibited the platelet aggregation activity.

#### 3.2.5. Coumarins

Three coumarins, siderin (**61**), 7-O-glucosyloxy-4-methoxy-5-methylcoumarin (**62**), 7-hydroxy-4-methoxy-5-methylcoumarin (**63**) have been isolated from *I. cylindrica*, and all the coumarins belong to simple coumarins, 4, 7-dimethoxy-5-methylcoumarin as a common scaffold. Among them, compound **61** has been proved to have antibacterial activity [23].

#### 3.2.6. Others

In addition to above-mentioned compounds, a sesquiterpenoid cylindrene (**66**) with vasoconstriction activity, was isolated again from rhizomes of this plant [17]. A phytotoxic tabanone (**69**) was isolated [20] to inhibit growth of frond area of duckweed, root growth of garden onion, and fresh weight gain of garden lettuce with 50% inhibition values of 0.094, 3.6, and 6.5 mM, respectively. The target site of tabanone remains unknown, but its mode of action results in rapid loss of membrane integrity and subsequent decrease in the rate of photosynthetic electron flow. The antimicrobial palmitic acid (**65**) and phytol (**68**) also have been isolated from *I. cylindrica*.

## 4. Progress of Pharmacological Studies on *I. cylindrica*

The traditional medicinal applications of *I. cylindrica* have inspired many pharmacological investigations. Extracts and isolated compounds from *I. cylindrica* plants exhibited multiple bioactivities—including diuretic activity, hemostasis, anti-inflammatory, antitumor, antibacterial, immunomodulatory. Here, we summarized the progress and list as follow.

### 4.1. Diuretic Activity

The diuretic activity of *I. cylindrica* plants was found to be related to their traditional uses for treating edema and difficulty in micturition. *I. cylindrica* water extract possessed diuretic activity in normal rabbit, the diuretic effect was the most obvious after administration for 5 to 10 days. This effect may be related to the nervous system, cutting off the peripheral nerves of the kidney, and its diuretic effect is lost. Additionally, the water extract can alleviate glomerular vasospasm, increase renal blood flow and renal filtration rate [24].

### 4.2. Hemostasis

The extract of *I. cylindrica* displayed hemostasis effect on rat blood heat hemorrhage model induced by dry yeast suspension combined with absolute ethanol. In addition, the aqueous extract of *I. cylindrica* can significantly shorten its prothrombin time, thrombin time and activated partial thromboplastin time, and its hemostasis is related to endogenous, exogenous thrombin, and internal and external sources. Other studies have shown that aqueous extract of *I. cylindrica* can enhance the activity of endogenous and exogenous coagulation system, up-regulated the expression of TXB2, reduced the level of 6-keto-PGF1α, promoted platelet aggregation, and enhanced hemostasis [25].

### 4.3. Anti-Inflammatory

Inflammation has been reported to be relative to various diseases. The aqueous extracts of *I. cylindrica* was reported to have anti-inflammatory activity by relieving the auricular edema in mice induced by dimethylbenzene, ameliorate the paw-swelling in rats by carrageenan, significantly blocking the increased permeability of celiac blood capillary by glacial acetic acid and was able to remarkably resist paw swelling in rats induced by zymosan A [26]. Some researchers studied the protective effect of *I. cylindrica* extract on renal tissues in rats with Adriamycin nephrosis. It was found that the ethyl acetate extracts of *Imperatae Rhizoma* had protective effect in rats with adriamycin nephrosis. It may be related to reducing the expression of NF-κB p65 and TGF-β1 and the content of TNF-α in renal tissues of rats reduce inflammation of kidney tissue [27].

In 2020, Putri et al. reported that the ethanol extract of cogongrass (*I. cylindrica* L.) roots could decrease the sepsis score and didnot cause any harm in body weight [28].

### 4.4. Antioxidant Activity

The good antioxidant capacity of *I. cylindrica* was found by Fenton method and potassium ferricyanide reduction method. Its IC_50_ for scavenging of hydroxyl radicals was 0.0948 mg/mL, while IC_50_ of ascorbic acid was 0.1096 mg/mL; in the ferricyanide considerable reduction method, the extract exhibited reducing power comparable to that of the ascorbic acid [29].

Other studies have shown that aqueous extract of *I. cylindrica* can enhance the activity of superoxide dismutase (SOD) in liver and brain tissue of mice with alcoholism, inhibiting the activity of hydroxyl radicals, reducing the level of malondialdehyde, improving the body’s antioxidant capacity, and alleviating the pathological damage of free radicals to liver and brain tissue. It suggested that *I. cylindrica* has a protective effect on liver and brain damage caused by alcoholism.

The hydroalcoholic root extract of *I. cylindrica* showed antioxidant activity of 14.33 ± 0.045 (10 µg/mL) to 36.56 ± 0.053 (50 µg/mL) by DPPH assay. Antioxidant activity was also confirmed with reduction by FRAP assays [30].

### 4.5. Antitumor

#### 4.5.1. Crude Extract

The aqueous extracts of *I. cylindrica* and polysaccharides can inhibit the growth of proliferation of human hepatoma cell line SMMC-7721, HepG2 cells and solid tumors in H22 mice. They also can increase the Interleukin (IL) 2 secretion level of leukocyte-mediated peripheral blood of tumor-bearing mice, suggesting that *I. cylindrica* extracts have anti-hepatic tumor effects. The different concentrations of *I. cylindrica* water extract were reported to dose-dependently suppress hepatoma SMMC-7721 cells. The mechanism of action is to increase S period ratio at the same time reduce the G2/M phase cell proportion, and inducing apoptosis of the cell [31]. In addition, the extract of *I. cylindrica* was proved to against cancer cells, when tested at 20 g/mL [32]. It was able to inhibit more that 50% the proliferation of the three tested cancer cells (MiaPaCa-2, CEM/ADR5000, CCRF-CEM). The lowest IC_50_ values of 6.86 g/mL on MiaPaCa-2 and 3.91 g/mL on CCRF-CEM cells were obtained with *X. aethiopica*, while the corresponding value of 6.56 g/mL was obtained with *P. capense* on CEM/ADR5000 cells. In addition, the extracts of *I. cylindrica* inhibited the proliferation of the tested cancer cell lines including sensitive and drug-resistant phenotypes [33]. The recorded IC50 ranges were 3.28 mg/mL (against HCT116 (p53^−/−^) cells) to 33.43 mg/mL (against HepG2 cells), and the extracts of *I. cylindrica* induced apoptosis in CCRF-CEM cells via the alteration loss of MMP whilst that of *Piper capense* also enhanced the production of ROS.

The *I. cylindrica* extracts also have anti-cancer effects on human oral squamous carcinoma cell line SCC-9 in vitro [34]. It treatment caused cytotoxicity and induced cell death in vitro in SCC-9 cells in a dose-dependent manner. Additionally, this treatment also significantly reduced the clonogenic potential and inhibited cell proliferation by arresting the cell cycle in the G2/M phase.

#### 4.5.2. Isolated Compounds

Arundoin (**1**) played a role in upregulating the expression levels of cleaved caspase-3, cleaved caspase-9 and Bax, downregulating Bcl-2, in a dose-dependent manner, indicated that arundoin can inhibit the proliferation of LNCap cells and induce apoptosis (Chen D.-K., 2017).Another research pointed that arundoin (**1**) can increase the cracking of polyadenosine diphosphate ribose polymerase(PARP) and block the proliferation of PC3 cells, promoting apoptosis of prostate cancer cell linePC3 [35]. 

2-Methoxyestrone (**13**) and tricin (**17**) exhibited considerable growth inhibitory activities against BT-549 and HT-29 cancer cell lines in vitro [9]. The half-maximal inhibitory concentration (IC_50_) of compounds **13** and **17** on BT-549 breast cancer cell line (72 h) was 102 µM and 68 µM, respectively. IC_50_ of compounds **13** and **17** on a HT-29 colon cancer cell line (72 h) was 147µM and 114 µM, respectively. Daucosterol (**6**) was reported to inhibit the proliferative ability of HL-7702, SMMC-7721, and HepG2 cells in a concentration-dependent manner, with IC_50_ values of 214.99, 143.40, and 138.73 µg/mL, respectively [36]. Therefore, these findings suggested that compound **6** significantly suppressed the proliferation ability of hepatocellular carcinoma (HCC) cells in a concentration-dependent manner. Besides, it was found that compound **6** inhibited the proliferation of human breast cancer cell line MCF-7 and gastric cancer cell lines MGC803, BGC823 and AGS in a dose-dependent manner. Furthermore, compound **6** inhibits murine hepatoma H22 cell growth in ICR mice. Treatment of compound **6** induced intracellular ROS generation and autophagy, but not apoptotic cell death [37].

### 4.6. Antibacterial

The extracts of *I. cylindrica* possessed antibacterial activity in inhibiting Quorum-sensing metabolites of Chromobacterium violaceum CV026 with antibacterial zone of 12 mm and Quorum-sensing inhibition zone of 20 mm; however, it showed no inhibition on *Pseudomonas aeruginosa* PA01 [38]. The different solvent extracts of *Imperatae rhizoma* possessed antibacterial activity in five tested strains. Among them, ethyl acetate extract had the best bacteriostatic effect on *Klebsiella aerogenes*, boiling extract against *Escherichia coli*, acetone extract for *Staphylococcus aureus*, boiled extract for *Bacillus subtilis*, and 50% ethanol extract for *Enterobacter aerogenes*, inhibition zone of 12.0, 13.0, 13.5, 13.5, and 15.0 mm, respectively.

### 4.7. Immunomodulatory

In the innate immunity, complement system is of significant importance. Components of complement can be activated sequentially by the classical pathway, the lectin pathway and the alternative pathway, the activated components of complement mainly mediate complement-dependent cytotoxicity to achieve clearance of pathogenic microorganisms and abnormal cells. It was found that the mineral ether extract of *I. rhizoma* and the ethyl acetate extract of *I. rhizoma* can reduce the activity of complement C1, C2, C4, C3, and C5 and inhibit the activation of the classical pathway of complement [7]. 

Macrophages are a subset of innate immune cells that the body mediates non-specific cellular immune responses, which exert direct phagocytosis and indirect secretion of cytokine-mediated immune regulation. NIH mice were orally administrated with *I. rhizoma* decoction up to 20th day, then 5% starch and 9% NaCl were intraperitoneally injected, after one hour, 1% chicken red blood cells were injected. It is found that the number of peritoneal macrophages, percentage of phagocytosis and phagocytic index were significantly increased. It suggests that *I. rhizoma* extract can enhance the non-specific immunity of the body. In addition, another research reported that water extract of *I. rhizoma* can inhibit the formation of nitric oxide in lipopolysaccharide-induced mouse macrophages RAW264.7 [39].

In the adaptive immune response, the immunomodulatory effects of *I. rhizoma* extract are mainly reflected in the subpopulation structure, proliferative capacity and secretion of related cytokines of T lymphocytes. Animal experiments showed that *I. rhizoma* decoction can increase the proportion of CD4+ T cells in immunodeficient mice, decrease the proportion of CD8+ T cells, adjust the ratio of CD +4 /CD8 + T cells to balance and restore their immune function. Meanwhile, *I. rhizoma* decoction can also increase the proportion of helper T cells (Th cells) in the spleen of NIH mice and increase the secretion of IL-2 [40].

### 4.8. Liver Protection Activity

The methanol extract of *I. rhizoma* can inhibit the damage of carbon tetrachloride to the liver without toxic side effects [6]. A report has shown that *Rhizoma Imperata* has a renal protective effect on the rat model of IgA nephropathy established by bovine serum albumin plus staphylococcal enterotoxin B method (Yin et al., 2011). Compared with the model group, URBC, 24hUP, Scr, BUN, PAF, and TGF-β1 expression were significantly decreased, while serum IL-2 was significantly increased (*p* < 0.01) in the treatment groups. It was suggested that *I. cylindrica* decoction can significantly reduce hematuria, proteinuria of the IgA nephropathy rats, reduce the pathological changes and improve renal function. The mechanism may be related to the elevation of IL -2 secretion of RI and inhibition of renal TGF-β1 secretion expression, etc.

### 4.9. Other Activity

The extract of the leaves of *I. cylindrica* possessed an antihypertensive action with a significant dose-dependent reduction in amplitude of contraction of smooth muscle cells of rabbit jejunum in comparison with standard antihypertensive drug, adrenaline [41]. The heart pressure of the cats was significantly reduced with 160 and 320 mg/mL of the extract from 266 to 180 mmHg (*p* = 0.012) but there was no effect on the heart rate. The minimum effective dose EC50 was 0.013. Ethanolic leaf extract of *I. cylindrica* exhibited vasodilative antihypertensive properties similar to mechanism of action of adrenaline. The extract could be used to manage hypertension.

Moreover, the extract of *I. cylindrica* played a role in regulating lipid metabolism and tolerance to hypoxia [42,43]. The polysaccharide of *Rhizoma Imperatae* can reduce the levels of glycated hemoglobin, triglyceride, total cholesterol, and low-density lipoprotein cholesterol in the serum of diabetic mice, increase the levels of hepatic glycogen and high-density lipoprotein cholesterol, and regulate the disorder of glucose and lipid metabolism. When mice were orally administered with *I. cylindrica* extract at the dose of 50, 100, 200, 400 mg/kg and propranolol at the dose of 20 mg/kg, the oxygen consumption was reduced respectively by 2.48 %, 23.14%, 10.74%, 5.79%, and 28.93% for 0 to 5 min, and 2 .11 %, 21.13%, 16.90%, 38.73%, and 9.86% for 5 to 10 min. The survival rates of mice with hypoxia for 30 min were 0, 0 ,20%, 10%, 50%, 20% when receiving saline, propranolol of 20 mg/ kg and RIP of 50, 100, 200, 400 mg·kg^−1^. The survival time was prolonged by 33.16%, 37.31%, and 35.23% in mice with hypoxia of propranolol and RIP of 200 and 400 mg/kg groups

## 5. Industrial Applications

Based on the record in “Compendium of material medica” and “ben jing”, *I. cylindrica* is considered to be effective in encouraging a variety of physical functions. These include stopping vomiting, improving renal function, increasing urination, clearing heat and toxins, relieving fever, and promoting hemostasis. Thus, it could be widely used for the treatment of fever and thirst, vomiting blood, epistaxis, pulmonary heat and asthma. Nowadays, a modern prescription “Qingre Tonglin Pian” has been approved by China Food and Drug Administration (Z20050124) for the hot-water drench treatment. This formula is mainly composed of an extract from *I. cylindrica* roots. *I. cylindrica* is utilized in other countries besides China. In India, it is one of the key ingredients in India’s various traditional medicinal formula and has multiple pharmacological applications. Generally, it is used as diuretic and an anti-inflammatory agent. Foodservice industry applications of *I. cylindrica* provide a novel choice for people to select health-care foods such as jellies, vinegar, beverages, sauces, flavoring agents, tea, or wines that contain some derivatives of *I. cylindrica* (Table 1). *I. cylindrica* is also used in the cosmetic industry. For instance, *I. cylindrica* root antibacterial powder is used to make natural non-toxic toothpaste. Furthermore, fermented liquor used for Chinese herbal compounds that contained *I. cylindrica* is used to make oil-control and acne-treatment cosmetic products. Selected patents regarding pharmacological applications of *I. cylindrica* is presented in Table 2.

## 6. Conclusions

In recent years, people have increasingly paid attention to the problems of multidrug resistance and side effects of drugs in existing drugs, forcing researchers and stakeholders to constantly try to develop safe and effective drugs. A wide variety of plants are traditionally and ethnopharmacologically used for treating various disorders, and some proved to be effective. Therefore, natural medicine is undoubtedly the best excavation point. The plant of *I. cylindrica* is an excellent medicinal plant with multiple bioactive constituents. The plant has been used for treating various diseases and extensive research has been initiated. Although various aspects of this plant are constantly making progress, the development and discovery of a new drug, even an allopathic phytomedicine from *I. cylindrica*, will require more detailed preliminary studies in both the preclinical and clinical venues.

The present review summarizes a comprehensive overview of the reported pharmacognosy, phytochemistry, and pharmacological activities of the plant *I. cylindrical*. Up to now, 72 compounds from *I. cylindrica* were identified, among them flavonoids and saponins are the main compositions. The reports on the bioactivities of extracts and compounds from *I. cylindrica* are chronicled, the main bioactive components are lignans and flavonoids, which may contribute either directly or indirectly to the biological effects of the *I. cylindrica* genus. Studies implemented through in vivo and in vitro experiments have demonstrated the bioactivities of *I. cylindrica* plants, most of which support their traditional medicine uses. Pharmacological studies are mainly focused on the anti-inflammatory, antitumor, antiviral and antioxidant activity, and liver protection activity. In addition, some studies indicated that it has immunomodulatory functions.

Nevertheless, some issues still remain unclarified. Firstly, the traditional diuretic and hemostasis actions of *I. cylindrica* require more modern pharmacology studies to clarify its underlying mechanism. As current activity studies mostly focus on in vitro, more in vivo studies and more clinical applications are needed to further optimize modern clinical drug standards. In addition, it should be noted that during our review, we observed that some studies have conflicting results, which suggest that these studies may not provide reproducible data. Moreover, study protocols were improperly described and there was heterogeneity in study protocols. These factors could hinder the replication of experiments. Strong scientific methodologies are required before confirmatory decisions can be made on the potential of *I. cylindrica.* Secondly, there are a limited number of active ingredients that have been pharmacological studies. Therefore, we need to answer if these identified substances can achieve the equivalent effect of *I. cylindrica*, or, if not, to what extent. Thirdly, the traditional using of TCM are commonly combined with other TCM in conventional therapies. The interactions and underlying mechanisms between *I. cylindrica* and other TCM should be further investigated by future studies. Furthermore, the current studies of this plant have primarily focused on its small molecule compounds, but have rarely investigated its macromolecular compounds. Thus, future studies of *I. cylindrica* could devote more effort to its macromolecular compounds, especially its polysaccharides. Fourthly, due to the complex composition of agents used in herbal medicine, a unified international identification of *I. cylindrica* is needed to control the quality of this herbal medicine and establishing a standardized fingerprint of this plant might be indispensable. Finally, although *I. cylindrica* is currently widely distributed and cultivated in many areas of the world, it is also important to manage the use of this plant and avoid overexploitation.

We believe that if we can solve the above questions in depth and reveal the medical potential of *I. cylindrica*, as well as other TCM, we can explore and utilize the precious medical reserve that TCM left to the human beings. Simultaneously, we hope this review highlights the importance of *I. cylindrica* and provides some directions for the future development of this plant.

## Figures and Tables

**Table 1:** Constituents isolated from *I. cylindrica*

NO.	Name	CAS	Formula	References
*Saponins*				
1	Arundoin	4555-56-0	C_31_H_52_O	[4]
2	Cylindrin	17904-55-1	C_31_H_52_O	[4]
3	26-Norolean-9(11)-ene, 3-methoxy-13-methyl-, (3β)-	2392-92-9	C_31_H_52_O	[4]
4	(8α)-Arborinol methyl ether	27570-19-0	C_31_H_52_O	[5]
5	β-Sitosterol	83-46-5	C_29_H_50_O	[6]
6	Daucosterol	474-58-8	C_35_H_60_O_6_	[6]
7	α-Amyrin	638-95-9	C_30_H_50_O	[6]
8	3-O-β-D-glucopyranosyl-6′-tetradecanoate	120000-27-3	C_49_H_86_O_7_	[6]
9	Friedelin	559-74-0	C_30_H_50_O	[7]
10	Alnusenone	508-09-8	C_30_H_48_O	[8]
11	Simiarenol	1615-94-7	C_30_H_50_O	[8]
12	Fernenol	4966-00-1	C_30_H_50_O	[8]
13	2-Methoxyestrone	362-08-3	C_19_H_24_O_3_	[9]
14	14-epiarbor-7-en-3β-ol	-	C_30_H_50_O	[10]
15	14-epiarbor-7-en-3β-yl formate	-	C_31_H_50_O_2_	[10]
16	14-epiarbor-7-en- 3-one	-	C_30_H_48_O	[10]
*Flavonoids*				
17	Tricin	520-32-1	C_17_H_14_O_7_	[6]
18	Caryatin	1486-66-4	C_17_H_14_O_7_	[6]
19	Jaceidin	10173-01-0	C_18_H_16_O_8_	[6]
20	4*H*-1-Benzopyran-4-one, 7-hydroxy-2-(4-hydroxy-3-methoxyphenyl)-3,5,6-trimethoxy-	58130-92-0	C_19_H_18_O_8_	[6]
21	5-Methoxyflavone	42079-78-7	C_16_H_12_O_3_	[7]
22	5-Hydroxyflavone	491-78-1	C_15_H_10_O_3_	[11]
23	Flavone	525-82-6	C_15_H_10_O_2_	[11]
24	2-(4-Hydroxyphenyl)-5-methoxy-4*H*-1-benzopyran-4-one	106848-87-7	C_16_H_12_O_4_	[11]
25	4*H*-1-Benzopyran-4-one, 6-(β-D-glucopyranosyloxy)-2-(4-methoxyphenyl)-	1588495-01-5	C_22_H_22_O_9_	[11]
26	5,7-Dihydroxy-8-methoxyflavone	632-85-9	C_16_H_12_O_5_	[11]
27	6-Hydroxy-5-methoxy-2-phenyl-4*H*-1-benzopyran-4-one	118021-60-6	C_16_H_12_O_4_	[11]
28	5-hydroxy-2-(2-phenylethyl) chromone	877673-99-9	C_17_H_14_O_3_	[12]
29	Flidersiachromone	61828-53-3	C_17_H_14_O_2_	[12]
30	5-Hydroxy-2-[2-(2-hydroxyphenyl)ethyl]-4*H*-1-benzopyran-4-one	357637-15-1	C_17_H_14_O_4_	[12]
31	5-Hydroxy-2-(2-phenylethenyl)-4*H*-1-benzopyran-4-one	389136-26-9	C_17_H_12_O_3_	[12]
32	Flindersiachromone	61828-53-3	C_17_H_14_O_2_	[11]
33	4*H*-1-Benzopyran-4-one, 5-hydroxy-2-(2-phenylethyl)-	877673-99-9	C_17_H_14_O_3_	[11]
34	8-Hydroxy-2-(2-phenylethyl)-4*H*-1-benzopyran-4-one	1588494-99-8	C_17_H_14_O_3_	[11]
35	4*H*-1-Benzopyran-4-one, 8-(β-D-glucopyranosyloxy)-2-(2-phenylethyl)	1588495-00-4	C_23_H_24_O_8_	[11]
36	Maritimin	3449-40-9	C_11_H_10_O_4_	[13]
*Glycosides*				
37	Salicin	138-52-3	C_13_H_18_O_7_	[8]
38	3,4-Dimethoxyphenyl 1-*O*-α-L-rhamnopyranosyl-(1→6)-*O*-β-D-glucopyranoside	872885-48-8	C_20_H_30_O_12_	[11]
39	Seguinoside K	257888-26-9	C_26_H_32_O_15_	[14]
40	Gymnetinoside D	1588477-87-5	C_27_H_34_O_15_	[14]
41	Deacetylimpecyloside	1622990-04-8	C_32_H_38_O_17_	[14]
42	Impecyloside	1070967-17-7	C_34_H_40_O_18_	[15]
*Phenols*				
43	Graminone B	161407-73-4	C_21_H_22_O_8_	[16]
44	Graminone A	161407-72-3	C_20_H_20_O_7_	[16]
45	Cylindol A	159225-89-5	C_16_H_14_O_7_	[17]
46	Cylindol B	159225-90-8	C_16_H_14_O_7_	[17]
47	Imperanene	163634-08-0	C_19_H_22_O_5_	[18]
48	4-Hydroxy-cinnamic acid	57-10-3	C_16_H_32_O_2_	[19]
49	Isovanillin	621-59-0	C_8_H_8_O_3_	[6]
50	Ethyl p-hydroxybenzoate	120-47-8	C_9_H_10_O_3_	[7]
51	Vanillic acid	121-34-6	C_8_H_8_O_4_	[7]
52	*p*-Vinylguaiacol	7786-61-0	C_9_H_10_O_2_	[20]
53	Protocatechuic acid	99-50-3	C_7_H_6_O_4_	[8]
54	1-*O*-*p*-Coumaroylglycerol	63529-09-9	C_12_H_14_O_5_	[8]
55	4-Hydroxybenzaldehyde	123-08-0	C_7_H_6_O_2_	[11]
56	Impecylone	1622990-03-7	C_14_H_14_O_4_	[14]
57	Impecylenolide	1622990-05-9	C_20_H_20_O_7_	[14]
58	Caffeic acid	331-39-5	C_9_H_8_O_4_	[13]
59	Ferulic acid	1135-24-6	C_10_H_10_O_4_	[13]
60	N-Acetyl-p-aminophenol	103-90-2	C_8_H_9_ NO_2_	[21]
*Coumarins*				
61	Siderin	53377-54-1	C_12_ H_12_ O_4_	[22]
62	7-*O*-Glucosyloxy-4-methoxy-5-methylcoumarin	41680-13-1	C_17_ H_20_ O_9_	[8]
63	7-Hydroxy-4-methoxy-5-methylcoumarin	41680-12-0	C_11_ H_10_ O_4_	[11]
*Other compounds*				
64	Dimethyl 4,4′-dimethoxy-5,6,5′,6′-dimethylenedioxybiphenyl-2,2′-dicarboxylate	73536-69-3	C_20_ H_18_ O_10_	[19]
65	Palmitic acid	57-10-3	C_16_ H_32_ O_2_	[19]
66	Cylindrene	158204-49-0	C_15_ H_20_ O_2_	[17]
67	5-Hydroxymethylfurfural	67-47-0	C_6_ H_6_ O_3_	[7]
68	Phytol	150-86-7	C_20_ H_40_ O	[20]
69	Tabanone	13215-88-8	C_13_ H_18_ O	[20]
70	3-(3,4,5-Trimethoxyphenoxy)-1,2-propanediol	68576-87-4	C_12_ H_18_ O_6_	[8]
71	3, 4-Dihydroxybutyric acid	1518-61-2	C_4_ H_8_ O_4_	[8]
72	L-Cysteine	52-90-4	C_3_ H_7_ NO_2_ S	[21]

**Table 2:** Patents list of products containing constituents from *I. cylindrical* and their claimed pharmacological properties

Application	Main Composition	Pharmacological Properties	Publish Number
Jelly	*I. cylindrica* roots, *Mume* fructus, *Mori* fructus*, Sophorae Flavescentis* radix, edible gelatin, Glyceryl Monostearate	Dispelling the effects of alcoholism	CN 107484997 A
Vinegar	*I. cylindrica*, *Lycium barbarum*, *Ziziphus jujube*, brewed rice vinegar with acidity of 3.5–4%	Relieving hangover and protecting the stomach	CN 109423430 A
Soft beverage	*I. cylindrica* roots, *Momordica grosvenori*, *Siraitia grosvenorii* fruits, *Chrysanthemum morifolium* flowers, crystal sugar	Clearing heat and detoxicating, cooling blood and hemostasis	CN 106418063 A
Solid beverage	sugar cane powder and *I. cylindrica* powder	Clearing heat and detoxicating, promoting urination, and salivation, relieving restlessness	CN 105495260 A
Sauce	*I. cylindrica*	Clearing heat and detoxicating, tonifying middle-jiao and qi	CN 104543941 A
Flavoring agents	*I. cylindrica* flowers, *Phyllanthus Emblica* fruits, antelope horn, *Panax Ginseng* leaves, edible vinegar essence, sesame oil, prebiotics	Tranquillizing heart and nourishing stomach	CN 106107858 A
Tea	*I. cylindrica* roots, *Paeonia suffruticosa* root barks, *Saposhnikovia divaricata* roots	Clearing heat and cooling blood, reinforcing disease resistance and preventing cold	CN 106689560 A
Tea	*I. cylindrica* root, *Lonicera japonica* flowers, crystal sugar	Clearing heat and toxic materials and relieving sore throat	CN 106689557 A
Wine	*Imperata cylindrical*, *Hedyotis diffusa*, *Semen Plantaginis*, 5 kg of pure grain spirit	Eliminating heat and toxin, removing dampness and treating stranguria	CN 103725482 A
Edible toothpaste	*Imperata cylindrica* root antibacterial powder, vitamin powder, fish gelatin	Preventing periodontal disease, antisepsis and containing no toxic chemicals	CN 106214600 A

## Data Availability

The data presented in this study are available on request from the corresponding author.

## Abbreviations

MIC	minimum inhibitory concentration
MMP	matrix metalloproteinase
MRSA	methicillin-resistant *Staphylococcus aureus*
NF-κB	nuclear factor-κB
ROS	reactive oxygen species
RSV	respiratory syncytial virus
SOD	superoxide dismutase
SsaA	staphylococcal secretory antigen
Syk	spleen tyrosine kinase
TBARS	thio-barbituric acid reactive substances
TCM	traditional Chinese medicine
TLR2	toll-like receptor
TNF-α	tumor necrosis factor alpha
TGF-β	transforming growth factor-β
UV	ultraviolet
